# Hemorrhagic Chemosis Associated With Isatuximab Use in an Elderly Lady With Multiple Myeloma

**DOI:** 10.7759/cureus.22764

**Published:** 2022-03-02

**Authors:** Precious O Idogun, Zeina Kayali

**Affiliations:** 1 Internal Medicine, Florida State University, Sarasota, USA

**Keywords:** premedication, monoclonal antibody, hemorrhagic chemosis, multiple myeloma, isatuximab, infusion reaction, target therapy oncology

## Abstract

Isatuximab is a newly approved targeted therapy for the treatment of patients with advanced multiple myeloma. Infusion reactions happen often with targeted therapies like isatuximab and can be severe or even life-threatening. However, ocular infusion reactions are rare. We report a 75-year-old female who presented with right eye erythema and associated pain and was found to have had a rapid onset of unilateral hemorrhagic chemosis following an initial infusion of isatuximab. She developed erythema in her right eye, associated with pain, swelling, burning, and a foreign body sensation. Visual acuity in the right eye decreased to light perception only within the first few minutes. The infusion was discontinued, and the patient was treated with steroids and intraocular pressure-lowering drugs. She was monitored for 24 hours and then discharged after symptomatic improvement.

## Introduction

Targeted/monoclonal antibody therapies have transformed the treatment landscape for cancer, producing remissions in tumors that were previously almost uniformly fatal [1]. Ocular adverse effects or infusion reactions from targeted therapies are relatively rare, but not unheard of. Ocular toxicities predominantly consist of uveitis, episcleritis, blepharitis, chorioretinopathy, and keratoconjunctivitis [1]. Isatuximab is a targeted monoclonal antibody that was approved for use by the Food and Drug Administration (FDA) on March 31, 2021 [2]. It is indicated for use in combination with carfilzomib and dexamethasone for adult patients with relapsed or refractory multiple myeloma who have received one to three prior lines of therapy [2]. The drug booklet notes that the most common side effects of Isatuximab include infusion reactions, leukopenia, anemia, thrombocytopenia, pneumonia, and diarrhea [3]. Given its relative novelty, it is important to monitor for adverse reactions that may have been absent or not reported during clinical trials.

## Case presentation

The patient is a 75-year-old female with a history of multiple myeloma (MM) with amyloidosis and light chain cast nephropathy, diagnosed four years prior to this presentation. She was presented to the emergency department with complaints of right eye erythema, pain, and swelling.

Since diagnosis with MM, the patient had initially been treated with a cyclophosphamide, bortezomib, and dexamethasone (CyBorD) regimen with no response. This regimen was subsequently changed to include daratumumab in combination with lenalidomide and dexamethasone. The patient was refractory to this treatment, so her regimen was changed to include ixazomib with pomalidomide and dexamethasone. Due to her inability to tolerate toxicities with this last regimen, the decision was made for her to start fourth-line therapy with isatuximab, carfilzomib, and dexamethasone. It is important to note that she did not receive carfilzomib on the day of her presentation to the hospital.

The patient had received a standard premedication regimen of 20 mg of dexamethasone and 50 mg of diphenhydramine. She then waited for one hour with no reaction before the initial infusion of isatuximab was started. Within 10 minutes, the patient developed rapid erythema of her right eye that was associated with pain, foreign body sensation, swelling, and burning sensation. There was proptosis of the right eye with a marked hemorrhagic response medially, inferiorly, and laterally. The left eye was unremarkable.

Visual acuity in her right eye decreased to light perception only. Following this development, the infusion was immediately discontinued, and a cold compress was applied. The patient was then transported to an ophthalmologist for further evaluation.

A physical examination at the ophthalmologist's office approximately 30 minutes after the infusion was stopped revealed that for the right eye, the patient was able to count fingers at 2 feet. Vision improved to 20/50+1 after 10 minutes and after receiving dexamethasone. For the left eye, visual acuity was 20/50-1. There was an afferent pupillary defect on the right side as well as a 2-3 mm proptosis in the right eye, which both resolved over a 10-minute period. Intraocular pressure in the right eye was 10 mm Hg, and in the left eye, it was 12 mm Hg. Fundoscopic examination demonstrated good retinal perfusion that improved over the same period of 10 minutes.

The patient was instructed by the ophthalmologist to present to the emergency department to further evaluate the possibility of extraosseous plasmacytoma, which may have become necrotic.

On admission to the hospital, initial vital signs were as follows: temperature of 97.3 °F, blood pressure of 103/61 mmHg, heart rate of 73 beats per minute. She was saturating 100% on room air and breathing at a rate of 18 breaths per minute.

**Figure 1:**
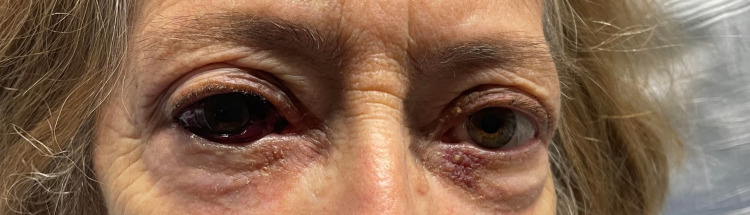
Proptosis and hemorrhagic chemosis of the right eye upon arrival to the hospital, approximately 10 hours after drug infusion was stopped.

**Figure 2:**
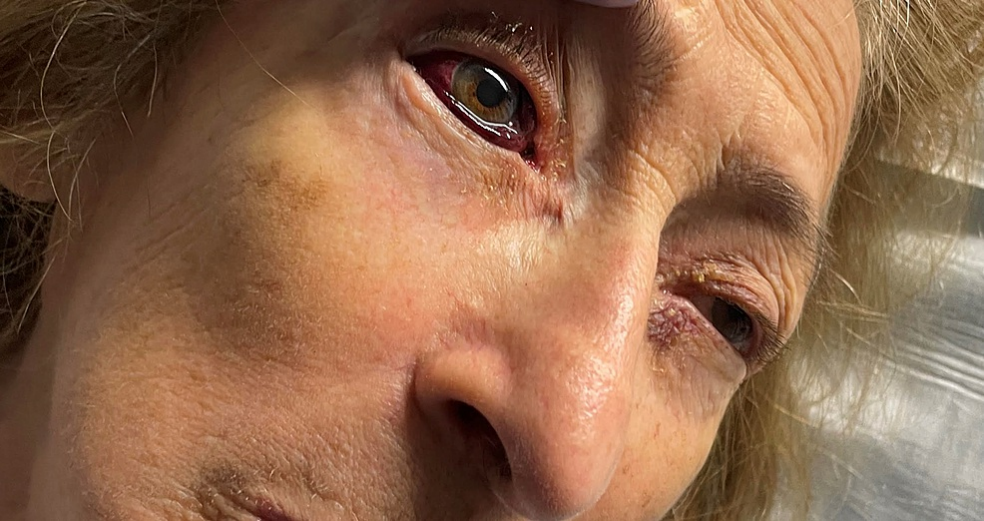
Hemorrhagic chemosis of the right eye, most evident inferiorly.

A physical examination at the time of hospital admission (approximately 10 hours from when the drug infusion was stopped) showed a well-appearing elderly female in no acute distress. The right eye examination showed proptosis and hemorrhagic chemosis (Figures 1-2). However, visual fields were intact, and pupils were equal and reactive. Visual acuity was measured at 20/50 bilaterally. Other components of the physical examination were largely unremarkable.

Investigations

The initial laboratory workup is summarized in Table 1. The complete blood count was significant for mild thrombocytopenia with a platelet count of 117 (150-400 × 10^3^/µL) which was the patient’s baseline. The basic metabolic panel demonstrated a GFR of 25 mL/min (>60 mL/min), which was consistent with her baseline CKD III. Other components of the laboratory workup were within normal limits.

**Table 1:** Serum chemistry and complete blood cell counts on admission. CBC: complete blood count, BMP: basic metabolic panel, GFR: glomerular filtration rate, WBC: white blood cells, RBC: red blood cells, MCV: mean corpuscular volume, MCH: mean corpuscular hemoglobin, MCHC: mean corpuscular hemoglobin concentration, RDW-SD: red cell distribution width-standard deviation, RDW-CV: red cell distribution width-coefficient of variation, MPV: mean platelet volume, NRBC: nucleated red blood cells, Meta: metamyelocytes, Myelo: myelocytes, Pro: promyelocytes, ANC: absolute neutrophil count, INR: international normalized ratio.

BMP		CBC			
Glucose (mg/dl)	286	WBC (×10^3^/µl)	4.6	RDW-CV (%)	13.7
Sodium serum (mmol/L)	139	RBC (×10^6^/µl)	3.69	Platelet (×10^3^/µL)	117 (150–400)
Potassium serum (mmol/L)	4.5	Hemoglobin (g/dl)	12.0	MPV (fL)	9.7
Chloride serum (mmol/L)	110	Hematocrit (%)	37.2	Segmented neutrophils (%)	87
Carbon dioxide serum (mmol/L)	23	MCV (fL)	100.8 (80–100)	Lymphocytes (%)	9
Blood urea nitrogen (mg/dl)	44	MCH (pg)	32.5	Monocytes (%)	3
Creatinine serum (mg/dl)	1.97	MCHC (g/dl)	32.3	Eosinophils (%)	0
Estimated GFR (ml/min)	25 (>60)	RDW-SD (fL)	50.5	Basophils (%)	0
Calcium serum (mg/dl)	9.1				

**Figure 3:**
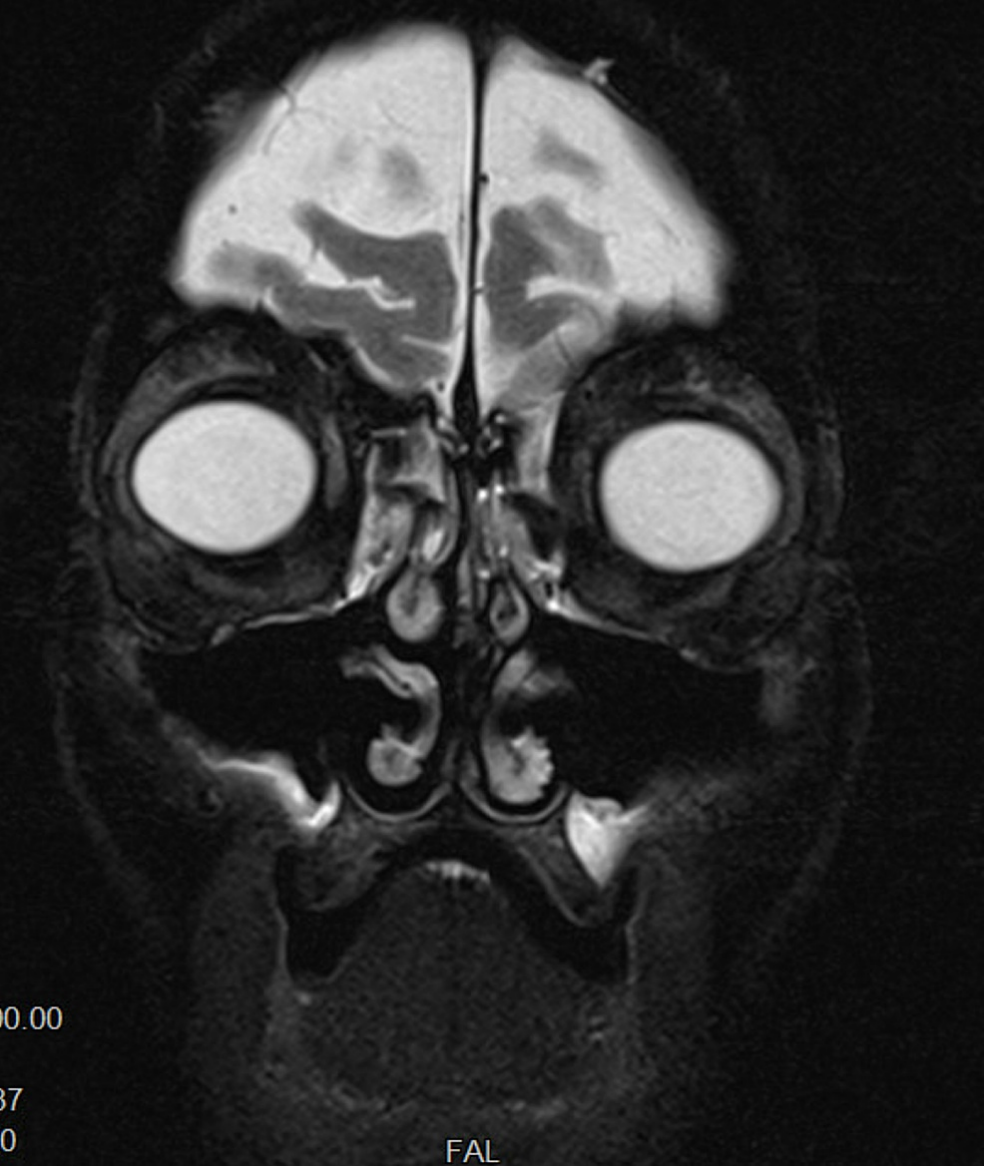
Coronal MRI view of the orbits.

Imaging was obtained, including computed tomography (CT) of the head and magnetic resonance imaging (MRI) of the orbits, as shown in Figure 3. The contrast was not used given the patient's poor renal function. CT of the head was evident for mild age-appropriate atrophic changes with stable ventricular size, no hemorrhage, and no midline shift. There was also evidence of pansinusitis with fluid. However, there was no acute intracranial process.

MRI of the orbits showed normal optic chiasm, prechiasmatic intracranial optic nerves, suprasellar cistern, and midline pituitary infundibulum pituitary gland. The right orbital soft tissues demonstrated the normal appearance of the globe and intraconal optic nerve sheath complex, with a normal appearance of the intraconal fat and retrobulbar structures. The superior ophthalmic venous vasculature has a normal flow void. The rectus musculature and orbital apex appeared normal as well. There were no extraconal lesions identified. The left orbital soft tissues were unremarkable.

Treatment

Once an infusion reaction was noted, the drug was immediately discontinued and a cold compress was applied to her eye. The patient then received 20 mg of dexamethasone in her oncologist’s office. Upon arrival at the ophthalmologist’s office, the patient received another 40 mg of dexamethasone intravenously. At the hospital, the patient was started on dorzolamide/timolol eye drops to lower intraocular pressure. She experienced substantial relief from her pain and swelling following these interventions. The patient was monitored in the hospital for 24 hours.

A physical examination prior to discharge showed visual acuity of 20/50 in the right eye and 20/50 in the left. She had 4+ subconjunctival hemorrhages for 360° around the right eye. Her proptosis had resolved and she had full extraocular movements. Anterior segment examination of the right eye at the bedside was unremarkable. Her pupil was reactive without an afferent pupillary defect.

Upon discharge from the hospital, she was advised to follow up with her outpatient oncologist within the next three days. The patient was subsequently maintained only on carfilzomib and cyclophosphamide. Isatuximab was permanently discontinued from her regimen.

## Discussion

The third most common hematologic cancer is multiple myeloma (MM) [4]. It is characterized by bone marrow infiltration with clonal plasma cells. Treatment of MM patients has radically changed over recent years following the introduction of next-generation proteasome inhibitors (PI) and immunomodulatory derivatives (IMiDs). Despite the presence of a variety of therapeutic modalities for the management of multiple myeloma, the introduction of monoclonal antibody therapy has changed the paradigm of the management of relapsed multiple myeloma [5]. Advances in molecular biology have led to the identification of aberrant proteins in cancer cells that are good targets for cancer therapy. Because these proteins are overexpressed or dysregulated in cancer cells compared with normal cells, the general assumption was that their inhibitors would be narrowly targeted and relatively nontoxic. However, this hope has not been accomplished.

Current targeted agents exhibit the same frequency and severity of toxicities as traditional cytotoxic agents, with the main difference being the nature of the toxic effects. Thus, the classical chemotherapy toxicities of alopecia, myelosuppression, mucositis, nausea, and vomiting have been generally replaced by vascular, dermatologic, endocrine, coagulopathic, immunologic, ocular, and pulmonary toxicities. Common side effects of various targeted therapies include mucosal irritation affecting any organ system. These toxicities need to be recognized, prevented, and optimally managed [1]. Their unique side-effect profile includes the potential for nonallergic infusion reactions caused by cytokine release [6]. They are also associated with infusion reactions.

Isatuximab is a CD38-directed cytolytic monoclonal antibody and is indicated in combination with dexamethasone and pomalidomide in the management of patients with multiple myeloma who have been previously treated with at least two prior agents, including a proteasome inhibitor and lenalidomide [3]. It is also indicated, in combination with dexamethasone and carfilzomib, for the management of adult patients with refractory or relapsed multiple myeloma who have been previously treated with one to three lines of therapy [3]. In this patient, her past treatments for MM included: cyclophosphamide, bortezomib, and dexamethasone, followed by daratumumab in combination with lenalidomide and dexamethasone, and finally ixazomib with pomalidomide and dexamethasone.

Infusion-related reactions (IRR) can be defined as "any signs or symptoms experienced by patients during the infusion of pharmacologic or biologic agents or any event occurring on the first day of drug administration" [7]. Combinations of IRR types may occur in the same patient [7]. They may present with constitutional symptoms such as fever, rigor, pruritus, hypotension, dyspnea, chest discomfort, rash, urticaria, angioedema, wheezing, or tachycardia, as well as the possibility of anaphylaxis requiring urgent intervention [8]. One of the more common side effects of isatuximab is infusion reactions.

Ocular adverse effects such as blurry vision and corneal disease have also been reported with different immunomodulatory agents in the management of multiple myelomas, such as belantamab mafodotin [9]. However, the literature is limited to the ocular adverse effects of isatuximab. To the best of our knowledge, this was the first case of hemorrhagic chemosis as a result of isatuximab use being reported. Furthermore, conjunctival side effects are rarely reported as ocular adverse effects with immunomodulatory treatment in multiple myeloma patients.

A baseline eye examination before treatment, followed by prompt referral to the ophthalmologist in the event of new complaints of potential symptoms such as red, painful, dry eyes, or visual disturbances, is recommended [10]. The eye may be susceptible to toxicity due to several factors, including its inherently robust blood supply, the presence of subpopulations of rapidly dividing cells, and an abundance and variety of cell surface receptors [11]. The severity of mAb-associated ocular toxicities is also variable, ranging from minor ocular irritation to severe vision-threatening events [11]. Overall, it is difficult to ascertain the incidence of ocular adverse effects in oncology patients, but they have been reported with increased frequency recently [10]. Furthermore, the mechanism of these adverse effects is largely undetermined.

## Conclusions

In this case, we present a patient who suffered an ocular infusion reaction to isatuximab despite being premedicated. Premedication does not always prevent adverse effects, and watchful monitoring for infusion reactions is required. There are also no well-established risk factors to identify patients more likely to suffer an acute infusion-related adverse event. Given its relative novelty, it is important to continue to monitor and document reactions of this kind to increase information and accurately inform potential recipients of the drug about the full safety profile and potential side effects of the medication. Ocular adverse effects from targeted therapy have been reported with increasing frequency, and it is imperative for medical and ocular oncologists to be cognizant of the spectrum of these side effects to effectively manage these complications.
